# Preoperative Mental Disorders and Hospital Healthcare Use in the First Year After Metabolic Bariatric Surgery: A Retrospective Study

**DOI:** 10.1007/s11695-025-07769-w

**Published:** 2025-03-05

**Authors:** Nadia Botros, Laura N. Deden, Elske M. van den Berg, Eric J. Hazebroek

**Affiliations:** 1 Vitalys Obesity Clinic, Part of Rijnstate Hospital, Arnhem, Netherlands; 2 Wageningen University &Amp, Research, Human Nutrition and Health, Wageningen, Netherlands; 3 Novarum Center for Eating Disorders and Obesity, Amstelveen, Netherlands

**Keywords:** Metabolic bariatric surgery, Mental disorders, Healthcare use

## Abstract

**Background:**

Mental disorders are relatively common in individuals who undergo metabolic bariatric surgery (MBS). Prior research suggests that mental disorders may relate to increased healthcare use after MBS. We retrospectively explored the association between preoperative mental health disorders and healthcare use in the first postoperative year.

**Methods:**

Patients who underwent primary MBS and had a structured preoperative psychological assessment report were included. Data on healthcare use was collected as the total number of non-routine healthcare appointments including inpatient, outpatient, and emergency department visits. Additionally, gastrointestinal (GI) healthcare use at the radiology, gastroenterology, and emergency departments was analyzed separately.

**Results:**

Of the 944 included patients, 261 (28%) had a preoperatively diagnosed mental disorder. Most prevalent were depressive disorders, anxiety disorders, and eating disorders. Patients with a preoperative mental disorder had a 15% (adjusted, CI 1.04–1.27, *p* = 0.005) higher rate of total healthcare use compared to those without. Among patients who had any GI-related healthcare, those with a mental disorder had a 61% higher rate of GI-related healthcare use (CI 1.02–2.55, *p* = 0.041). Patients with a mental disorder tended to have 20% lower odds of having no GI-related healthcare appointments (unadjusted, not statistically significant, CI 0.37–1.74, *p* = 0.568).

**Conclusion:**

The presence of preoperative mental disorders was weakly related to higher total non-routine hospital healthcare use in the first year after MBS. Models explained only 5–13% of the variation in appointment frequency, meaning unmeasured and/or unknown factors play a role in healthcare use.

## Introduction

Mental disorders are relatively common in individuals who undergo metabolic bariatric surgery (MBS), with a prevalence of 23% compared to 12–14% in healthy weight or general populations [1–3]. Most prevalent mental disorders in individuals undergoing MBS are depressive disorders (19%) and binge eating disorders (17%) [1].

To date, it is not fully understood to what extent preoperative mental disorders influence postoperative physical and mental health outcomes. It is, for example, unclear whether the incidence or severity of physical complaints after MBS are associated with mental disorders. Postoperative physical complaints such as gastrointestinal (GI) problems, including abdominal pain, are reported by 34–54% of patients who underwent MBS [4–6]. Patients with mental disorders may be at higher risk of experiencing postoperative complaints and pain. Postoperative pain has been related to (preoperative) depression and anxiety symptoms in other types of surgery [7, 8]. Anxiety and depression symptoms have been related to pain within the first 24 h after MBS [9]. Additionally, the presence of depressive and/or anxiety symptoms was found to be associated with GI symptoms in general and primary care populations [10–12] and has been suggested to be bidirectionally associated with IBS and functional dyspepsia, although this requires further investigation [13].

GI complaints and postoperative pain are expected to result in more healthcare use. Therefore, the aim of this study was to retrospectively explore the association between preoperative mental disorders and non-routine healthcare use during the first postoperative year in patients undergoing MBS. This exploration can contribute to better understanding of postoperative outcomes in patients with and without mental disorders, ultimately informing better patient management strategies.

## Materials and Methods

### Study Design and Population

In this retrospective observational study, medical files were used to collect data on diagnoses of preoperative mental disorders and healthcare use in the first year after MBS. The study procedures were approved by the institutional research committee of Rijnstate Hospital (Arnhem, the Netherlands).

Patients were included if they underwent a primary Roux-en-Y gastric bypass (RYGB) or sleeve gastrectomy (SG) at Vitalys obesity clinic (part of Rijnstate hospital) at least a year ago at the time of data collection (surgery between April 2020 and November 2022) and when a complete structured preoperative psychological assessment report was available. Patients were excluded if they underwent a secondary MBS procedure or if the presence of mental disorders during the assessment was uncertain (e.g., “potentially”).

Patients were divided in two groups based on preoperative presence of DSM-5 mental disorders [14], yes or no, which was assessed by a psychologist. Mental health status was assessed through DSM-5 based semi-structured interviews. The psychologists’ structured reports indicated the presence of eating disorders, depression, anxiety disorders, personality disorders, attention deficit (hyperactivity) disorders (AD(H)D), mild mental disability, or alcohol and substance abuse.

### Data Collection

Data collection was facilitated by CTcue (CTcue B.V., Amsterdam, the Netherlands, v4.11.1), a search engine for medical files.

#### Patient Characteristics

Data on patient characteristics, including age, gender, anthropometrics, smoking status, comorbidities and history of abdominal surgery, type of surgical procedure, type of support program, and postoperative complications within 30 days, were collected. The following comorbidities at presurgical screening were retrieved: diabetes mellitus type I or II (DM), cardiovascular disease (CVD, ICD-10 codes: I00-I99), obstructive sleep apnea (OSA), gastroesophageal reflux disease (GERD), inflammatory bowel disorder (IBD), irritable bowel syndrome (IBS), and fibromyalgia.

#### Healthcare Use

Data on healthcare use was collected as the number of non-routine appointments at the departments of MBS, gastroenterology, and radiology (GI-related) and GI-related emergency department visits at Rijnstate hospital/Vitalys obesity clinic during the first postoperative year. The following routine appointments were excluded: multidisciplinary support program sessions, standard medical follow-up, and appointments with the psychologist. The latter appointments were excluded as these are frequently planned if patients have mental disorders.

Analyses were done for total healthcare use (A) and more specifically for GI-related healthcare use (B). Total healthcare use was defined as all non-routine appointments with physicians, nurses, or dieticians: face-to-face/telephone/video consultations, abdominal diagnostic tests (gastroscopies, colonoscopies, computed tomography’s, echography’s, barium swallow tests, gastric emptying scintigraphy’s, magnetic resonance imaging), and GI-related emergency department visits. GI-related healthcare use was defined as GI-related healthcare at the gastroenterology, radiology, and emergency departments (including the previously mentioned abdominal diagnostic tests).

#### Body Weight Change

Body weight change at 12 months (± 90 days) after surgery was assessed as body mass index (BMI) change (BMI at follow-up − BMI at surgery) and the percentage of total body weight loss (%TWL) (weight loss at follow-up / body weight at surgery) × 100).

### Data Analysis

Descriptive analysis of patient characteristics was performed in the total population and stratified for prevalent preoperative mental disorders (yes or no); expressed in means ± SD, medians (Q1; Q3) or *n* (%). Differences in patient characteristics between those with and without mental disorders were determined with *χ*^*2*^ tests or Fisher’s exact tests for categorical variables and independent samples *t* tests or Mann–Whitney-Wilcoxon tests for continuous variables.

The association between mental disorders and total healthcare use and GI-related healthcare use was evaluated using negative binomial regression, providing incidence rate ratio’s (IRR)(95% CI). For GI-related healthcare use, the zero-inflated variant of negative binomial regression was used to take the excess zero’s into account, as the majority of the study population had no GI-related appointments. The count models give the IRR’s in patients with any GI-related appointments, and the zero models provide the odds ratio’s (ORs) for having zero GI-related appointments.

Potential confounders and covariates were selected based on literature and on the data. Assessed potential confounders or covariates were age, gender, surgery type, program type (group or individual), preoperative BMI categories (< 40 and ≥ 40 kg/m^2^), TWL% categories (per 10 units), DM, CVD, GERD, OSA, fibromyalgia, IBD, IBS, history of abdominal surgery, smoking status, and short-term complications. To select confounders and covariates, associations with both mental disorders and healthcare use were determined. The models with the lowest Akaike information criterion (AIC) were selected. For total healthcare use, the following additional variables were included in the model: IBS, GERD, DM, CVD, surgery type, pre-operative BMI, and gender. For GI-related healthcare use, this was IBS and GERD.

Data was analyzed using R and RStudio (RStudio, Inc., Boston, version 2023.12.1.402, and R version 4.3.0, R foundation, 2020, Vienna, Austria). *P*-values < 0.05 were considered to be statistically significant.

## Results

Within the defined period, 1862 patients who underwent a primary RYGB or SG were assessed for eligibility. Of those, 827 (44%) patients had no structured psychological assessment report, and in 91 (8%) patients, psychological status was unclear or inconclusive. This resulted in a total sample size of 944 patients.

Of these 944 included patients, 261 (28%) were diagnosed with one or more preoperative mental disorder. Patient characteristics are shown in Table 1. Anxiety disorders (*n* = 75, 29%), depressive disorders (*n* = 75, 29%), and eating disorders (*n* = 66, 25%) were most prevalent among patients with preoperative mental disorders. Patients with mental disorders were significantly younger, were more frequently involved in an individual MBS support program instead of a group-based support program, more often had IBS in their medical history, and had slightly higher BMI change and %TWL 12 months after surgery as compared to patients without mental disorders. In both groups, patients attended the routine appointments of the support program and medical follow-up similarly often.

**Table 1:** Patient characteristics in patients with and without mental disorders

	Overall (*n* = 944)	Mental disorder		
		No (*n* = 683)	Yes (*n* = 261)	*p*-value
Gender: female (*n* (%))	725 (76.8%)	523 (76.6%)	202 (77.4%)	0.856
Age (years)	46.2 ± 12.2	47.2 ± 12.0	43.9 ± 12.3	** < *0.001***
Surgery type (*n* (%))				0.843
RYGB	721 (76.4%)	520 (76.1%)	201 (77.0%)	
GS	223 (23.6%)	163 (23.9%)	60 (23.0%)	
Pre-operative BMI (kg/m^2^)	42.7 ± 5.12	42.7 ± 5.19	42.9 ± 4.96	0.471
BMI change at 12 months	−14.6 ± 3.74	−14.4 ± 3.74	−15.1 ± 3.68	***0.011***
Missing	149 (15.8%)	104 (15.2%)	45 (17.2%)	
%TWL at 12 months	34.2 ± 7.60	33.8 ± 7.57	35.3 ± 7.62	***0.014***
Missing	149 (15.8%)	104 (15.2%)	45 (17.2%)	
Support program type: group (*n* (%))	836 (88.6%)	624 (91.4%)	212 (81.2%)	** < *0.001***
Medical follow-up consultations	1.00 [1.00; 2.00]	1.00 [1.00; 2.00]	1.00 [1.00; 2.00]	0.474
Pre-surgery program sessions (max. 5)	5.0 [5.0; 5.0]	5.0 [4.5; 5.0]	5.0 [5.0; 5.0]	***0.002***
Post-surgery program sessions (max. 10)	8.00 [6.00; 9.00]	8.00 [6.00; 9.00]	8.00 [6.00; 9.00]	0.741
Extra consultations with psychologist	0 [0, 1.00]	0 [0, 1.00]	0 [0, 1.00]	** < *0.001***
GERD (*n*, (%))	142 (15.0%)	95 (13.9%)	47 (18.0%)	0.141
IBS (*n* (%))	72 (7.6%)	44 (6.4%)	28 (10.7%)	***0.037***
IBD (*n* (%))	15 (1.6%)	10 (1.5%)	5 (1.9%)	0.573
DM (*n*, (%))	191 (20.2%)	140 (20.5%)	51 (19.5%)	0.813
CVD (*n* (%))	374 (39.6%)	283 (41.4%)	91 (34.9%)	0.077
OSA (*n* (%))	190 (20.1%)	138 (20.2%)	52 (19.9%)	0.995
Short-term complications (*n*, (%))	29 (3.1%)	20 (2.9%)	9 (3.4%)	0.839
Previous abdominal surgery (*n*, (%))	360 (38.1%)	274 (40.1%)	86 (33.0%)	0.051
Fibromyalgia (*n* (%))	51 (5.4%)	34 (5.0%)	17 (6.5%)	0.412
Missing	27 (2.9%)	17 (2.5%)	10 (3.8%)	
Smoking status (*n* (%))				0.325
Current	98 (10.4%)	66 (9.7%)	32 (12.3%)	
Former	406 (43.0%)	290 (42.5%)	116 (44.4%)	
Never	440 (46.6%)	327 (47.9%)	113 (43.3%)	
DSM-5 axis (*n* (%))				-
Axis 1	186 (19.7%)	N.A	186 (71.3%)	
Axis 2	75 (7.9%)	N.A	75 (28.7%)	
Depressive disorder (*n* (%))	75 (7.9%)	N.A	75 (28.7%)	-
Anxiety disorder (*n* (%))	75 (7.9%)	N.A	75 (28.7%)	-
Eating disorder (*n* (%))	66 (7.0%)	N.A	66 (25.3%)	-
Personality disorder (*n* (%))	52 (5.5%)	N.A	52 (19.9%)	-
AD(H)D (*n* (%))	48 (5.1%)	N.A	48 (18.4%)	-
Mild mental disability (*n* (%))	25 (2.6%)	N.A	25 (9.6%)	-
Substance or alcohol abuse (*n* (%))	12 (1.3%)	N.A	12 (4.6%)	-

*BMI* body mass index, *%TWL* percentage total weight loss, *GERD* gastroesophageal reflux disease, *OSA* obstructive sleep apnea, *IBD* inflammatory bowel disorder, *DM* diabetes mellitus, *CVD* cardiovascular disease, *IBS* irritable bowel syndrome, *DSM* Diagnostic and Statistical Manual of Mental Disorders, *AD(H)D* attention-deficit (hyperactivity) disorders, *N.A.* not applicable

**Fig. 1:**
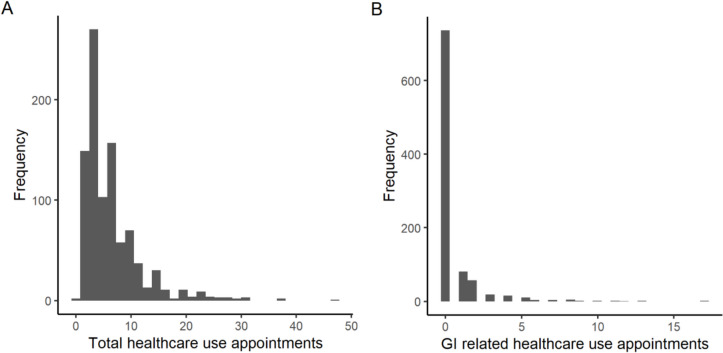
Histograms of first year non-routine healthcare use (**A** total, **B** gastrointestinal-related)

**Fig. 2:**
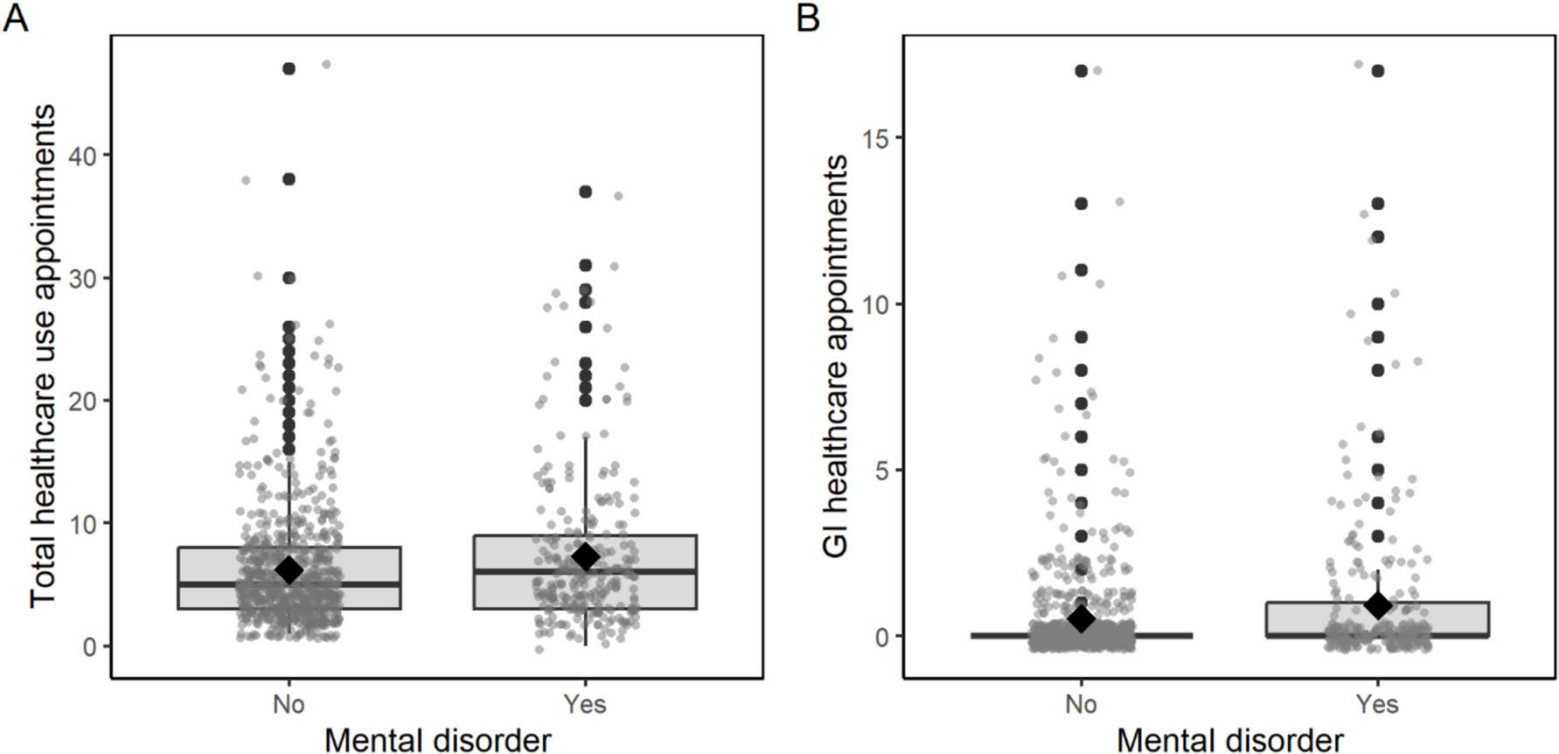
Boxplots of first year non-routine healthcare use (**A** total, **B** gastrointestinal-related) by the presence of preoperative mental health disorders

The distribution of non-routine healthcare appointments is shown in Fig. 1. Figure 2 shows boxplots of healthcare use by the presence of preoperative mental disorders. The median number of non-routine total healthcare use appointments was 5 [3; 8] in those without mental disorders and 6 [3; 9] in patients with mental disorders (Table 2). Twenty-two percent of the population (*n* = 208) had any GI-related healthcare use. In the group of patients with mental disorders, this was 27%, compared to 20% in the group without mental disorders (*p* = 0.035).

**Table 2:** First postoperative year non-planned healthcare use by mental disorders

	Mental health disorders		Overall (*n* = 944)
	No (*n* = 683)	Yes (*n* = 261)	
A. Total healthcare use frequency^a^	5.00 [3.00; 8.00]	6.00 [3.00; 9.00]	5.00 [3.00; 8.00]
B. GI-related healthcare use frequency^b^	0 [0; 0]	0 [0; 1.00]	0 [0; 0]
Any GI-related healthcare use: yes (*n*, %)	138 (20.2%)	70 (26.8%)	208 (22.0%)

^a^All non-routine appointments with physicians, nurses, or dieticians at the departments of MBS, gastroenterology, radiology, and gastrointestinal-related care at the emergency department

^b^All appointments at the gastroenterology, radiology, and emergency departments (gastrointestinal-related)

The crude and adjusted (zero-inflated) negative binomial regression analyses are shown in Table 3. The adjusted incidence rate ratio (IRR) for total healthcare use in patients with mental disorders compared to those without was 1.15 (CI 1.04–1.27, *p* = 0.005, pseudo *R*^2^ 0.05). In patients that did have any GI-related healthcare use, the IRR for having GI-related healthcare was 1.61 (CI 1.02–2.55, *p* = 0.041, *R*^2^ 0.09) for patients with mental disorders compared to those without (*count model*). After adding IBS and GERD to the model, the IRR changed to 1.57 (CI 1.00–2.48, *p* = 0.052, *R*^2^ 0.13). The odds ratio (OR) for having zero GI-related healthcare appointments was 0.80 (CI 0.37–1.74, *p* = 0.568) if individuals had mental disorders (*zero model*). The OR changed to 0.90 after adding IBS and GERD to the model.

**Table 3:** (Zero-inflated) Negative binomial regression models of non-planned healthcare use in 944 patients in the first year after MBS

		Beta	SE	IRR^a^	95% CI LL	95% CI UL	*p*-value	(Pseudo) *R*^2^
A. Total healthcare use								
Crude	(Intercept)	1.82	0.03	6.19	5.87	6.53	0.000	0.01
	Mental disorder: yes	**0.16**	**0.05**	**1.18**	**1.07**	**1.30**	**0.001**	
Adjusted model	(Intercept)	1.86	0.05	6.39	5.76	7.10	0.000	0.05
	Mental disorder: yes	**0.14**	**0.05**	**1.15**	**1.04**	**1.27**	**0.005**	
	IBS	0.18	0.08	1.20	1.02	1.42	0.029	
	GERD	0.15	0.06	1.16	1.03	1.32	0.017	
	DM: yes	0.19	0.06	1.20	1.08	1.35	0.001	
	CVD: yes	− 0.11	0.05	0.90	0.82	0.99	0.027	
	Surgery type: SG	0.16	0.05	1.17	1.06	1.30	0.003	
	Pre-operative BMI: ≥ 40	− 0.12	0.05	0.89	0.80	0.98	0.018	
	Gender: male	− 0.09	0.06	0.91	0.82	1.02	0.094	
B. Gastrointestinal-related healthcare use								
Crude								0.09
* Count model*	(Intercept)	− 0.08	0.40	0.92	0.42	2.02	0.8374	
	Mental disorder: yes	**0.48**	**0.23**	**1.61**	**1.02**	**2.55**	**0.041**	
* Zero model*	(Intercept)	− 0.26	0.90	0.77	0.13	4.47	0.772	
	Mental disorder: yes	** − 0.23**	**0.40**	**0.80**^**a**^	**0.37**	**1.74**	**0.568**	
Adjusted model								0.13
* Count model*	(Intercept)	− 0.03	0.32	0.97	0.51	1.83	0.921	
	Mental disorder: yes	**0.45**	**0.23**	**1.57**	**1.00**	**2.48**	**0.052**	
	IBS: yes	0.20	0.32	1.22	0.64	2.30	0.546	
	GERD: yes	0.14	0.28	1.15	0.66	2.00	0.626	
* Zero model*	(Intercept)	0.08	0.61	1.08	0.33	3.54	0.899	
	Mental disorder: yes	** − 0.10**	**0.35**	**0.90**^**a**^	**0.45**	**1.80**	**0.770**	
	IBS: yes	− 1.62	1.61	0.20^**a**^	0.01	4.62	0.313	
	GERD: yes	− 0.37	0.48	0.69^**a**^	0.27	1.75	0.433	

*SE* standard error, *IRR* incidence rate ratio, *CI* confidence interval, *LL* lower limit, *UL* upper limit, *IBS* irritable bowel syndrome, *GERD* gastroesophageal reflux disease, *DM* diabetes mellitus, *CVD* cardiovascular disease, *SG* sleeve gastrectomy, *BMI* body mass index

^a^Odds ratio (OR) in zero models

## Discussion

In this study, the association between preoperative mental disorders and non-routine hospital healthcare use in the first year after MBS was assessed. Healthcare use was used as a proxy for postoperative complaints. The findings in this study indicate that preoperative mental disorders were weakly positively associated with higher non-routine healthcare use in the first year after MBS. The appointment rate was 15–18% higher for non-routine healthcare and in patients with any GI-related healthcare use the rate of GI-related healthcare use was 57–61% higher (the latter was borderline significant after adding IBS and GERD to the model). Patients with mental disorders tended to have 10–20% lower odds of having zero GI-related healthcare appointments; this was not statistically significant. It should be noted that the used models only explained 5–13% of the variation in healthcare appointments, meaning unmeasured and/or unknown factors play a major role in healthcare use (e.g., individual factors, surgery-related factors, or dietary factors).

The current study suggests a weak association between mental disorders and healthcare use after MBS. Previously, only few studies have reported on healthcare use and psychological factors after MBS. In a population with chronic diseases (*N* = 991,445), mental disorders were associated with substantially higher healthcare use [15]. In a cohort study in the USA (*N* = 8192), patients with mental disorders had 20–40% lower odds of having zero all cause emergency department visits in the first year after MBS, depending on mental disorder severity [16]. Among the patients that did have emergency department visits, those with a mental disorder had a 40–70% higher IRR for 1-year emergency department visits [16]. Our study finds a similar trend for GI-related healthcare use, with a 10–20% lower odds ratio for having no GI-related healthcare (not statistically significant) and 57–61% higher IRR in case of any GI-related appointments.

Several studies have described associations between mental health and healthcare use, postoperative pain, or GI complaints. Research so far has focused mostly on anxiety and depression symptoms. In a prospective cohort study by Kvalem et al. [17], preoperative anxiety symptoms were associated with a higher perceived impact of several somatic symptoms three years after MBS (*n* = 163) [17]. In other studies, depression and anxiety symptoms were related to pain within the first 24 h after MBS [9] and to postoperative pain in other types of surgery [7, 8]. In contrast, another study (*n* = 160) found that depression and anxiety symptoms were not related to chronic abdominal pain 5 years after MBS [6]. Next to postoperative pain, the association between mental disorders and GI-related healthcare may partially be explained by the relationship between the brain and the gastrointestinal tract. Reviews, for example, suggest bidirectional links between depression and anxiety symptoms and GI-related diseases or problems such as IBD, IBS, and functional dyspepsia, although this still needs to be further elucidated [13, 18]. Earlier, depressive and/or anxiety symptoms were associated with GI symptoms in general and primary care populations [10–12].

This study adds to the understanding of postoperative healthcare use of patients with preoperative mental disorders undergoing MBS. Although the found association is weak, it is interesting to further investigate more direct measurements of GI symptoms instead of healthcare use. It can be useful to further study these associations in patients with co-occurring GI disorders or symptoms. Eventually, this knowledge may lead improved personalized therapies to minimize adverse health outcomes. These therapies may include cognitive behavioral therapy, nutritional therapy, and/or medication [18, 19].

This study has several strengths: to our knowledge, this is one of the few studies investigating mental disorders and healthcare use after MBS. Mental health conditions were assessed by psychologists during semi-structured interviews, which strengthens the quality of the data on psychological status.

Several limitations have to be noted as well. Only healthcare use at the hospital where MBS was performed was recorded, and (except for emergency department visits) individual reasons for non-routine appointments were not collected. Therefore, actual healthcare use is underestimated. In addition, some factors, such as IBD and short-term complications, could not be used in models as their occurrence in the study population was too low for providing reliable estimates. Moreover, due to the sample size restrictions, we could not investigate the association with different mental disorders separately. Lastly, concerning generalizability, there can be cultural, national, or institutional differences that influence healthcare use. Haddad et al., for example, report that almost 70% of their patients underwent non-routine postoperative imaging [20]. In our study population, 22% received non-routine GI-related care, which besides abdominal imaging included consultations as well.

## Conclusion

Preoperative mental disorders were weakly related to higher non-routine hospital healthcare use in the first year after MBS. Among patients using gastrointestinal-related healthcare, those with mental disorders had significantly more gastrointestinal-related appointments than those without. Patients with mental disorders tended to have lower odds of having no gastrointestinal-related appointments, although this finding lacked statistical significance. Models could only explain a minor proportion of the variation in appointment frequency, meaning unmeasured and/or unknown factors play a major role in healthcare use.

## Data Availability

No datasets were generated or analysed during the current study.
